# The Kaiona Framework: Centering Hawaiian and Pasifika Community in Defining, Measuring, and Promoting Health and Well-Being

**DOI:** 10.3390/ijerph23030402

**Published:** 2026-03-22

**Authors:** Kenny S. Ferenchak, Blane K. Garcia, J. Kukui Maunakea-Forth, Chelsey V. Jay, Isaiah Pule, Eric Enos, Kay L. Fukuda, Asia Engle, C. Kamalani Cruz, Myna Keleb, Angelica Raza-Furtado, Alika Spahn Naihe, Andrew Aoki, Faith Ewaliko, Uʻilani O. N. Schnackenberg, Kevin M. C. D. Akiyama, Ariel Makana Panui, Kyle Kaliko Chang, May Okihiro

**Affiliations:** 1 Waianae Coast Comprehensive Health Center, Waiʻanae, HI 96792, USA; blanekc@hawaii.edu (B.K.G.); okihirom@hawaii.edu (M.O.); 2 MAʻO Organic Farms, Waiʻanae, HI 96792, USA; 3 Hoa ʻĀina O Mākaha, Waiʻanae, HI 96792, USA; 4 E Ala Voyaging Academy, Waiʻanae, HI 96792, USA; 5 Kaʻala Farm, Waiʻanae, HI 96792, USA; 6 Student Equity, Excellence and Diversity (SEED) Department, University of Hawaiʻi at Mānoa, Honolulu, HI 96822, USA; 7 Place-Based Afterschool Literacy Support (PALS), Honolulu, HI 96822, USA; 8 Theorycraftist Games, Waiʻanae, HI 96792, USA; 9 Islander Institute, Kailua, HI 96734, USA; 10 ʻElepaio Social Services, Waiʻanae, HI 96792, USA; 11 Department of Pediatrics, John A. Burns School of Medicine, University of Hawaiʻi, Honolulu, HI 96813, USA

**Keywords:** indigenous health, youth mental health, community-based participatory research, health equity, community empowerment, community-informed intervention, cultural relevance

## Abstract

**Highlights:**

**Public health relevance—How does this work relate to a public health issue?**

- This work represents a community response to youth mental health and chronic health conditions, critical issues to the health systems and populations of Hawaiʻi, the USA, and the globe.
- This work aims to address the structural problems that are at the root of health disparities faced by our Indigenous community.

**Public health significance—Why is this work of significance to public health?**

- Under the direction of community leaders and rooted in place-based traditions and culture, this work details the effort of a Hawaiian community to make clear its own priorities and values around health and well-being, directly in response to the outside systems and metrics that have failed to uplift the gifts and abundance of this place.
- The Kaiona Framework detailed in this work serves as the foundation for subsequent efforts of our collective: (a) creating novel quantitative and qualitative methodologies to assess health status and program impact based on local traditions and values; (b) formalizing and assessing a referral system for pediatric patients in our local community health center to culture-based programming to address mental health and chronic health needs; and (c) catalyzing partnerships across the healthcare, education, and nonprofit sectors in our community to improve the system of care for community youth.

**Public health implications—What are the key implications or messages for practitioners, policy makers and/or researchers in public health?**

- Investing the time and effort to listen to authentic community voices may reveal bold, creative, and impactful solutions to critical problems such as youth mental health and chronic health conditions that are already operating in communities.
- In prioritizing the values, traditions, assets, and leaders unique to a place, broader systems such as healthcare, education, and the nonprofit sector can tailor public health interventions to communities.

**Abstract:**

The place and people of Waiʻanae, Hawaiʻi, are rich in connection with ʻ*āina* (natural environment) and culture. Counter to this strengths-based approach, metrics and narratives imposed by outside systems assess many communities like ours as “sick”, “poor”, or “unwell”. This paper details our community’s approach to defining “well-being” around the values specific to our place, overseen by a council of community leaders with decades of experience supporting youth. The development was a mixed methods process including formal focus groups, informal community conversations, review of existing models, and collaboration with a professional artist. Centering community was the priority through each phase, engaging youth, parents, cultural practitioners, healthcare providers, and educators. Our community built the Kaiona Framework around the *moʻolelo* (traditional story) of Kaiona who helps the lost find home through empathy and compassion. Well-being is grounded in connection to, in relationship with, and in service to *ʻāina*. The child is at the center of our work, but inseparable from the family, community, and wider nation of people. Wellness comprises four values vital to our community: *mauli ola*, a balanced state of physical, mental, emotional, spiritual, and environmental health; *waiwai*, abundance and prosperity; *pilina*, mutually sustaining relationships; and *ea*, self-determination and agency.

## 1. Introduction

“He lokomaikaʻi ka manu o Kaiona.

*Kind is the bird of Kaiona.* Said of one who helps a lost person find their way home. The goddess Kaiona, who lived in the Waiʻanae Mountains of Oʻahu, was said to have pet birds who could guide anyone lost in the forest back to their companions

([1], #770).”

In 2023, the National Institutes of Health (NIH) Common Fund launched an initiative to develop, share, and evaluate community-led health equity structural interventions that leverage partnerships across multiple sectors to reduce health disparities across the United States [2]. Waiʻanae Coast Comprehensive Health Center (WCCHC), one of the oldest and largest Federally Qualified Health Centers in the State of Hawaiʻi, is one of 15 community-based organizations (CBOs) nationally awarded funding to conduct structural intervention projects to change the social, physical, economic, and/or political environments that shape the health of our community.

Waiʻanae is a Hawaiian community of abundance. This wealth exists in the forms of proud *Kānaka Maoli* (Native Hawaiian) and *Pasifika* (Pacific Islander) identity and traditions, a rich natural environment, and strong family bonds. Much of this is captured in a deep sense of connectedness by which individuals are intimately tied to their extended families, broader community, *ʻāina* (natural environment), and spirituality. This wealth also exists in the forms of powerful community organizations and influxes of resources into the community. Yet, Waiʻanae faces health, educational, economic, and social struggles, now exacerbated to critical levels after the COVID-19 pandemic. Youth adolescent and mental health needs in Waiʻanae are representative of broader trends that have been declared a national emergency [3].

The Hawaiian view of self extends beyond the individual to the family, community, natural world, and spiritual realm [4]. Rather than the individual, *ʻohana*, or the extended family, has been described as the basic social unit [5]. Traditionally, *ʻohana* includes the present family system, past connections to ancestors that involve land and genealogy, and linkages to future generations through the perpetuation of language and other cultural practices [5]. Consequently, one’s wellness cannot be limited to the individual’s physical or mental state. Personal health is more accurately captured by the concept of *lōkahi*, harmony and balance between *kanaka* (humankind), *ʻāina*, and *akua* (God or gods) [5]. A more accurate representation of health has been described as an “Ecological Model of Native Hawaiian Wellbeing” which incorporates ʻĀina Wellbeing, Nation Wellbeing, Community Wellbeing, ʻOhana Wellbeing, and Individual Wellbeing [5]. For centuries, these perspectives served as the foundations for a prospering Indigenous civilization across the islands of Hawaiʻi. Likewise, Hawaiian medicine and healing practices like *lāʻau lapaʻau* and *hoʻoponopono* were premised upon maintaining the harmonious interdependence of the individual, extended family, elements of nature, and spiritual world [6]. Mental health was every bit as important to wellness as physical health, and physical ailments could only be addressed after the successful treatment of psychic issues.

Traditional Hawaiian views of self and wellness do not always align with modern efforts to address mental health needs. The Euro-North American lens that frames much of our healthcare system today is based upon siloed specialization and individual treatment [7]. Despite the guise of objectivity, reviews of decades of psychotherapy treatment data establish the extent to which current best practices have been based on Eurocentric ideas and populations; any data on non-white subjects are exceedingly rare [8]. Furthermore, most mental health problems have been described, characterized, and measured in the setting of Western high-income societies; thus even identifying problems in diverse communities is a challenge with the lack of properly validated screening instruments [9]. When directly compared to standard interventions, culturally adapted psychotherapies have been shown to be significantly more effective for non-white populations [10,11,12]. As if the philosophical disconnect were not enough, modern mental health issues in Hawaiʻi are rooted in the very concrete history of cultural repression, political oppression, economic marginalization, and environmental degradation Hawaiians have lived through in the past two-and-a-half centuries since first European contact [13]. Native Hawaiian youth have been found to suffer an undue burden of mental illness [14], adversity [15], and family struggles [16] compared to non-Hawaiians, and community providers have noted the COVID-19 pandemic to only worsen the mental and behavioral health struggles faced across the Waiʻanae community.

Given the modern crisis in youth mental health and research in recent decades making clear the interplay of individual biological and broader environmental factors, even the highest authorities within the Euro-North American medical establishment now recognize the promise of a more holistic approach [17]. The holistic view of health has been demonstrated to determine the individual’s overall sense of well-being in the Hawaiian community [18]. Naturally, then, emotional and psychological well-being can be enhanced via interventions that focus on extended family and relationships, culture and ethnic identity, and connection to the natural world [15,16,18,19,20].

Some have suggested, however, that evidence supporting the effectiveness of these approaches is lacking. For one, no standard exists to gauge the depth of cultural integration into programs [21]. This means some interventions claiming to be rooted in Hawaiian culture may only incorporate surface-level elements like Hawaiian terms or activities whereas deep structure requires intimate appreciation of Hawaiian heritage and beliefs. Inappropriate use of culture can leave programs ineffective or even harmful to participants given the reactions that can occur [13]. Additionally, assertions of the cultural and spiritual nature of Native Hawaiian culture-based programs can extend into defenses that efficacy cannot be measured by conventional Western measures [21]. As a result, personal testimony and anecdotes are often the only data offered in program evaluations.

As Hawaiian communities have demonstrated remarkable resilience over centuries of struggle since the time of contact, this moment may similarly present a moment of opportunity.

**Figure 1:**
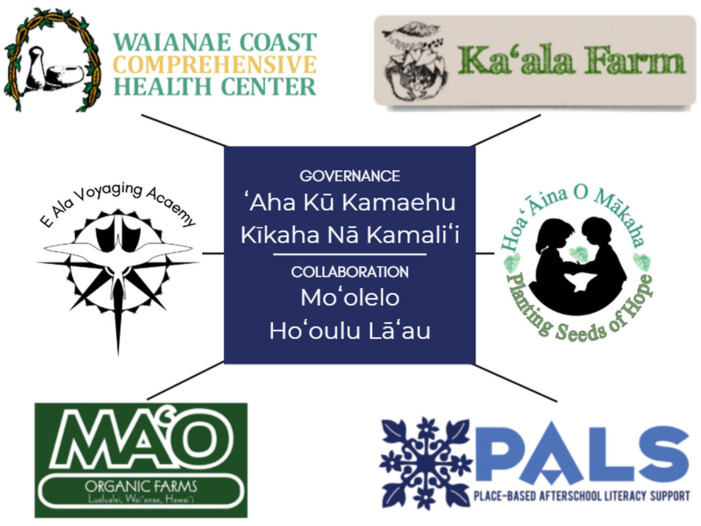
Collective governance. In this partnership between the community health center and 5 community-based organizations, we prioritize governance, ensuring that multigenerational voices of our community are directing the work. ʻAha Kū Kamaehu (community council); Kīkaha Nā Kamaliʻi (youth council); Moʻolelo (storytelling); Hoʻoulu Lāʻau (enhancing existing work).

WCCHC’s project, Kamaehu o ke Kaiāulu, aims to foster community connections through *Kānaka Maoli* cultural values to strengthen youth health and well-being along the Waiʻanae Coast. This leeward coast of the island of Oʻahu is home to one of the largest *Kānaka Maoli* communities in the world. *Kamaehu* (firmness of resolution) signifies our commitment to the community, particularly in bringing forth the *ehu* (first beam of sunlight) inherent in each *kama* (child). Waiʻanae is known for its intense heat, yet it is cooled by the gentle *kaiāulu* wind specific to this place, allowing for vital work to be done. *Kaiāulu* is also the Hawaiian word for “community.” Drawing strength from this *kaiāulu* wind, both the breeze and the collective spirit of its community, we in Waiʻanae confront modern health, economic, social, and environmental challenges with empathy, compassion, and *aloha* (love). We envision a community of support that empowers every child to realize their gift and have a path to live that gift [22]. ʻAha Kū Kamaehu (ʻAha Kū) is the governing collective of this work, convening the leadership of five community-based organizations (CBOs) and representatives of WCCHC (see Figure 1).

As one of our first collective efforts, it was important to assess the perspectives of our community on well-being, health, and resilience. Our place and our people are rich in our connection with *ʻāina*, strong family ties, cultural identity, and resilience in the face of adversity. However, too many communities like ours have been labeled “sick”, “poor”, or “unwell” because of metrics imposed by outside systems and values. Those very systems are often the profit-driven, ecologically destructive/extractive, and culturally disconnected forces that are prone to perpetuating cultural, social, and economic inequities. We reflected on our personal and community stories to develop an approach that validates our experience and celebrates our values.

## 2. Materials and Methods

The ʻAha Kū community council oversaw the methods described here through formal monthly meetings and other regular communications. In initial meetings, the council decided that the first step in the Kamaehu effort was to specify the intended outcome of the work, thus leading to the decision to pursue a community-specific perspective on health and well-being. Community leaders cited the wisdom of traditional Polynesian navigators in identifying a destination as the initial step in planning a voyage. When this decision was made, Kamaehu investigators were simultaneously conducting a community scan as prescribed by the NIH, a mixed methods study consisting of focus groups, a review of internal health center data, an analysis of partner organization program evaluations, and a survey of existing data sources around topics of holistic health in Hawaiʻi. All of that research helped steer the framework development.

Guided by the work of other Indigenous communities and researchers, we have sought to utilize *moʻolelo* (storytelling) as a core method in decolonizing and indigenizing health research [23]. Storytelling serves a culturally relevant and respectful method of centering community voices that can double as a therapeutic healing process in and of itself. Coupling storytelling with other methods of data gathering, we aimed to adopt a “two-seeing perspective” that respects both Indigenous and Western ways of knowing as legitimate sources of understanding, rooting in a “both–and” perspective as opposed to an either–or mandate [24].

Centering on *moʻolelo* entailed recognition of the many forms that stories take in our community. To weave together past, present, and future, we anchor our work in *ʻike kūpuna* (ancestral wisdom). This includes the *ʻōlelo noʻeau* (traditional Hawaiian proverbs) that we use to open this manuscript, as we did to begin each ʻAha Kū meeting and focus group. This practice was an invaluable method of rooting our work in acknowledgement that all we do is built upon those who came before us and is in service to the future generations.

Another rich form of *moʻolelo* abundant in our community is “talk story”, an informal approach to conversation. Free-flowing conversations in the community, side discussions at our ʻAha Kū meetings, and long-winding debriefing sessions guided framework development in invaluable ways. Though not recorded or analyzed like focus groups or research interviews, we approached these informal sources of *moʻolelo* in ways appropriate to both our local norms and standard Western research practice.

Formal interview and focus group participants represented a diverse cross-section of the community, including *ʻōpio* (youth), *mākua* (parents), *mahi ʻai* (farmers), and WCCHC healthcare providers. A total of 24 community members contributed to the formal portion of this study. Of the 24 participants, the majority were from the Waiʻanae community and ranged in age from 17 to 56 years. The majority (68%) identified as Native Hawaiian. Thematic analysis was conducted to analyze the transcripts. Research team members followed a thorough two-step qualitative coding process to identify key themes and sub-themes. The analysis prioritized cultural approaches to acknowledge the importance of interwoven relationships between community, individuals, culture, and well-being.

General trends in mental health and chronic health diagnoses were analyzed in the electronic medical records (EMR) of our community health center. The EMR database analyzed consisted of 36,634 individuals, aged 1 day to 25 years, who were seen at WCCHC from 2018 to 2023. Among the patients, 21.8% were 20–25 years of age, 23.6% were 13–19 years of age, 43.8% were Native Hawaiian, 18.3% were other Pacific Islander, 13.6% were Filipino, 53.1% identified as female at birth, and 46.9% identified as male. The majority (83.7%) were economically disadvantaged as indicated by enrollment in Hawaiʻi’s Quest Medicaid program.

Per the ʻAha Kū community council’s directive to define health and well-being, Kamaehu investigators performed a literature review of holistic frameworks of health and well-being. Investigators based their review on approaches encountered at various conferences and through other professional experiences, seeking frameworks particularly relevant to youth and Indigenous communities built around outcomes of true value, beyond just disease onset, morbidity, and mortality.

## 3. Results

Honoring the commitment to local governance and the traditions of our place, the methods described above were conducted concurrently, with results from various components continuously being interwoven and shared with the ʻAha Kū community council as they guided the overall direction of this process.

### 3.1. ʻAha Kū Guidance on Framework Development

Table 1 summarizes a variety of frameworks identified outside of Hawaiʻi along with a brief rationale behind their relevance to the local context of the Waiʻanae community. The literature review and conversations with colleagues from around the region brought to the fore several relevant frameworks created with similar intentions around Hawaiʻi, outlined in Table 2. The research team presented these existing models to the ʻAha Kū council, suggesting the possibility of adopting one of the frameworks to guide the Kamaehu project. A series of informal and formal meetings were held to foster discussion across CBOs and among leaders representing different generations. In small groups, council members reviewed existing frameworks under the “I like, I wish, I wonder” format whereby advantages, weaknesses, and points of needed clarification were identified for each model (see Table 2).

The council quickly reached unanimous consensus in opting to develop a new framework specific to our place and our community rather than adopting an outside model. Community leaders of ʻAha Kū acknowledged the richness embedded within each of the existing models, particularly those developed in other areas across Hawaiʻi. However, the individuals and organizations comprising ʻAha Kū know intimately the long history of outside models, programs, and resources coming to our coast with oftentimes disappointing results. ʻAha Kū thus felt strongly that this investment by the NIH necessitated local, place-based leadership in all phases, particularly this critical first step of defining health and well-being on our own terms.

**Figure 2:**
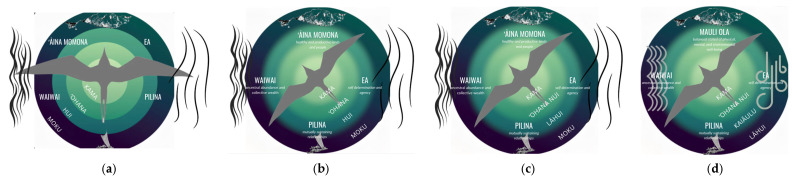
Evolution of the Kaiona Framework. (**a**) Draft 1 based on ʻAha Kū community council conversations, pulling from existing frameworks across Hawaiʻi while incorporating imagery and elements specific to the Waiʻanae community. (**b**) Draft 2, based on community leader feedback, reorients the central ʻ*iwa* figure to direct to the northwest (see Section 4) and to honor the four cardinal directions, while adding definitions for the four key cultural values. (**c**) Draft 3, based on community feedback, redefines the layers of community, ʻ*ohana* (family) to ʻ*ohana nui* (extended family) emphasizing the importance of extended family beyond blood relations, and *hui* (club, organization) to *lāhui* (nation, people) to be more inclusive. (**d**) Draft 4, based on ongoing community conversations and ʻAha Kū reflections, substitutes the domain *mauli ola* for ʻ*āina momona* as a better conceptualization of holistic health. The layers of community are further refined to have *kaiāulu* (community) and *lāhui* (nation, people) as the outer bands. Feedback on imagery specifically requested that the elemental symbols for water and wind align better with Polynesian traditions.

Under this new directive, the research team summarized the findings of the review of existing models and surrounding conversations into guiding principles for the development of a Waiʻanae-specific framework (see Table 3). The broader ʻAha Kū reviewed this document and contributed feedback that ultimately led to the drafting of a well-being framework based on the *moʻolelo* of Kaiona. This draft image went through multiple rounds of revision (see Figure 2) with a series of informal and formal feedback sessions with ʻAha Kū, again utilizing the “I like, I wish, I wonder” format. Later draft versions were also shared with youth leaders nominated by each of the CBOs within ʻAha Kū to gather their input.

Once the draft framework image and description reached a point of collective acceptance from ʻAha Kū, members nominated a professional artist and cultural practitioner based on his prior experience with multiple CBOs of ʻAha Kū. The group worked with the artist in a collaborative, creative, and iterative exploration and conceptualization based on the draft documents to produce versions of a finalized Kaiona Framework. He presented these at a daylong retreat of the collective ʻAha Kū where members provided minor recommendations on imagery and ultimately reached firm universal consensus on moving forward with the Kaiona Framework presented below.

### 3.2. Synthesis of Focus Group Analysis, Moʻolelo, and Data Review

Formal analysis of the focus groups revealed the following themes: community connectedness, youth health, healing, generational differences, culture and identity, and ideal “dream” programs. These themes were introduced throughout the ʻAha Kū proceedings described above, enriched by the multiple forms of *moʻolelo* encountered through the process as well as the research team’s review of internal health center data and existing data sources around topics of holistic health in Hawaiʻi. A strengths-based approach highlighted the assets of the community while also revealing challenges and opportunities, ultimately aligning with the major domains that comprise the Kaiona Framework.

#### 3.2.1. Health and Wellbeing

Community assets made clear in this domain included connection to *ʻāina*, the holistic conception of balance known as *lōkahi*, and the rich tradition of *ʻāina*-based organizations promoting wellness. Conversely, well documented health disparities amongst *Kānaka Maoli* and Pacific Islander youth matched our internal EMR data, specifically in terms of chronic conditions such as asthma, obesity, and diabetes.

#### 3.2.2. Resources and Abundance

Culture came through as an anchor of the community, a source of great pride and also a source of holistic healing, framing Waiʻanae as a site of cultural abundance. On the other hand, in terms of modern economic metrics, real challenges came through with Waiʻanae facing some of the highest rates of poverty and houselessness in the state [39], exacerbated by the crippling cost of living on the island [40].

#### 3.2.3. Connection and Relationships

Community connectedness, multigenerational family culture, and connection to place came through as major strengths of the Waiʻanae community. A major challenge identified for community youth was a sense of feeling lost, a condition that did not fully align with conventional mental health diagnoses like depression in our EMR data.

#### 3.2.4. Self-Determination and Agency

Individual reflections across generations confirmed the reputation of Waiʻanae as a fiercely independent community, respected for its collective ability to defend from outside influences and chart its own course, embodied by the renowned CBOs of Waiʻanae serving youth and *ʻāina* for decades. Acknowledging formal educational attainment as a very imperfect proxy, particularly for a community like Waiʻanae given the historic and ongoing forces of colonialism and economic marginalization within the educational system, students have long struggled at Waiʻanae’s schools, scoring lower on various educational achievement tests, as well as attending and completing college at lower rates compared with others across the State [41].

## 4. Discussion

Throughout the framework development process described above, the project investigators worked with ʻAha Kū members in authoring a text summary of the perspective, values, and imagery that served as the foundation for the Kaiona Framework. That text served as the foundation of this Discussion section.

**Figure 3:**
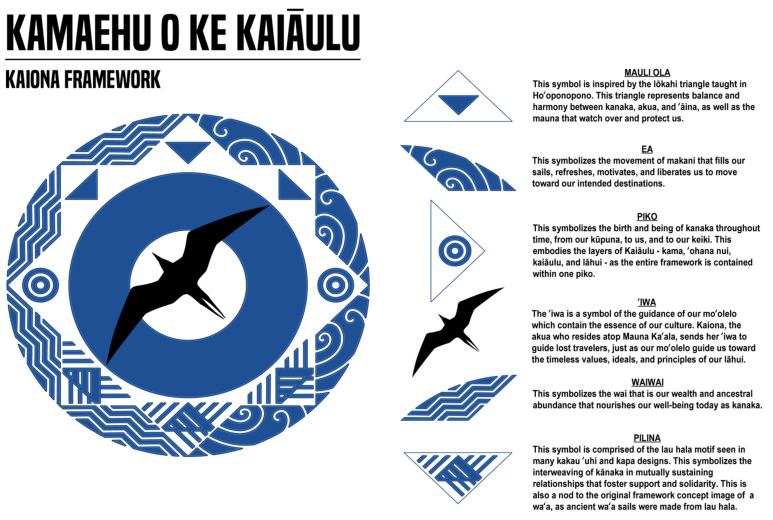
The framework imagery in its entirety is a representation of navigation through both space and time. The 8-pointed star surrounding the central point represents the 8 cardinal directions. To the left in the western region is the *waiwai* symbol, representing the ancestral abundance of our past. To the right in the eastern region is the *ea* symbol, representing the winds of emerging times that breathe life into our daily activities and endeavors. The *lōkahi* triangle representing *mauli ola* in the northern region repeats 3 times to denote harmony within the past, present, and future. The *lau hala* triangle representing *pilina* in the southern region repeats 3 times to denote relationship and support in the past, present, and future. The *piko* symbol in the west triangle represents the *kūpuna* that have come before us and the *piko* symbol in the east represents the *keiki* (children) who have yet to emerge. The large *piko* that contains the entirety of the framework represents us, as *lāhui*, in the present and for all time, for all time is now. Finally, the *ʻiwa* is a direct nod to the star navigational compass of Oceania, facing northwest toward our origin of our journey as a people, as well as toward Kaʻala for us on Oʻahu.

The Kaiona Framework validates our experience and celebrates our values of the Waiʻanae community (see Figure 3).

### 4.1. Framework Elements

#### 4.1.1. ʻIwa (Great Frigatebird)

*Moʻolelo* is the foundation. The framework calls people *home*, back to themselves, back to their identity; that is the essence of this work. Invoking Kaiona, who resides at *Mauna Kaʻala* (tallest point on Oʻahu, located in the Waiʻanae Range), the *ʻiwa* immediately establishes that this framework belongs to the Waiʻanae *moku* (region). Through *aloha*, empathy, compassion, and generosity, we will together find the destination—our home, our identity, our well-being on *our* terms. The *ʻiwa* signifies that spirituality is at the core. Oriented toward the northwest, we look to our ancestors, grateful for the *ʻike kupuna* (ancestral knowledge) that will guide all we do. The directionality also nods to the focus of our work being the Waiʻanae Coast of Oʻahu. Lastly, we acknowledge the resemblance to the Hawaiian star compass, honoring the direct role traditional voyaging and navigation has in our community and also the star compass itself as a model of Indigenous technology thriving in the modern context [42].

#### 4.1.2. Place

We honor our place and the community-based organizations (whose leadership comprises the ʻAha Kū community council) rooted in our place. *Mauna Kaʻala*, our sacred mountain, is the highest peak of our island, the home of so much spiritual force and source of so much life, referenced in the triangle at the top of the framework. Kaʻala Farms similarly serves as the source from which so much of our modern day *ʻāina*-based work flows, establishing a model of reclaiming and preserving the living culture of the *Poʻe Kahiko* (people of old) in order to strengthen the kinship relationships between the *ʻāina* and all forms of life necessary to sustain the balance of life [43]. For generations Hoa ʻĀina o Mākaha has been a beacon of hope and joy, creating peaceful communities in harmony with nature through the eyes, hands, and hearts of our children [44]. Through an unwavering commitment to the incredible wealth of spiritual, cultural, and intellectual capital in our Waiʻanae community, PALS enriches the experience of students by connecting them with that wealth, instilling a love for their place and their people, and fostering a commitment for the present and a heart for the generations yet to come [45]. MAʻO Organic Farms serves as a model of social enterprise, connecting youth and land through the daily practice of *aloha ʻāina*, empowering youth to succeed in college and secure sustaining careers and growing organic produce that yields individual and communal vitality [46]. The restoration of E Ala, the traditional voyaging canoe of our community that was among the first to be built during the modern renaissance of Polynesian voyaging, along with other vessels like Hōkūleʻa, embodies the powerful timing of our current efforts for this community. The weaving imagery of *lau hala* at the bottom of the framework represents the sails of these canoes. E Ala Voyaging Academy aims to empower and unite our community through the preservation and celebration of traditional Hawaiian practices, while fostering a supportive environment that instills a deeper sense of *kuleana* (responsibility and privilege) to our *ʻāina* and *kai* (ocean) [47]. It is the belief in, commitment to, and practice of *aloha ʻāina* that continues to bind these intimately connected organizations.

#### 4.1.3. Connection: Wai (Water) and Makani (Wind)

*Mauka* (mountain) to *makai* (sea), fresh water at the left of the framework is the natural connection that runs through our community and gives life. We tap into the natural order that has guided Hawaiian civilization since ancient times and also honor the contemporary work of our leaders who brought the water back at Kaʻala Farms and in doing so inspired the *ʻāina*-based work that thrives in our community today. In the intense heat of our coast, it is said that the gentle *kaiāulu* wind specific to this place is what allows for necessary work to be done, represented at the right of the framework. As the Hawaiian word for “community,” *kaiāulu* also signifies the strength of its people, who with empathy, compassion, and *aloha* together confront modern economic, social, and environmental challenges.

#### 4.1.4. ʻĀina

Mountain and ocean, water and wind—the natural elements surround our work, as illustrated by the outer ring of the framework. Western perspectives view land and the natural environment as geographic areas that can be owned, controlled, and sold. Many Pasifika and Indigenous communities, including *Kānaka Maoli*, see and appreciate land as a key part of their identity. This explains why Indigenous peoples take great pride in their upbringing. Land is not just land alone, but an integral part of who we are. Rather than ownership, stewardship. We honor reciprocal relationships with ancestral homelands. We recognize that it is *ʻāina* that heals, teaches, sustains, and gives life to us all. It is our *kuleana*, our great responsibility and our great privilege, to preserve, protect, and serve this *ʻāina.*

#### 4.1.5. Layers of Kaiāulu

We conceive of health and well-being as something beyond merely an individual. These layers all deserve attention in their own right, but there are no hard borders where one layer ends and another begins. The framework depicts these layers with the *piko* (navel) symbol, capturing sacred connections between past, present, and future, while the concentric circles also illustrate the levels of community that must be considered. *Kama* (child) is the heart of our community, both in that the individual child will be a focus of our efforts and that ultimately the most meaningful work is that which is designed for our future generations. Our children depend upon their *ʻohana nui* (extended family), celebrating the strength of family that so richly defines our community while also calling attention to modern forces such as addiction, poverty, and incarceration that have strained strict blood relations. Our community likewise prides itself in a collective identity of *kaiāulu*, maintaining a deep pride in being from this place of resilience and strength. And while focused on this *moku*, we recognize the *kuleana* that this work will ultimately impact the broader *lāhui* (nation).

#### 4.1.6. Values of Kaiāulu

- ***Mauli ola*** sits atop the framework as we work for the balanced state of physical, mental, and environmental well-being, a holistic health for the individual and the collective. Occupying the north as a cardinal direction, *mauli ola* can be seen as our *Hoku Paʻa* or North Star, the fixed point guiding our efforts. The triangle imagery references Mauna Kaʻala, stressing that well-being is directly connected to *ʻāina*, and the proximity to the *ʻiwa* reminds us that spirituality is an essential component of health and well-being. These all come together in the *lōkahi* triangle, the critical connection between spirituality, land, and people.
- ***Waiwai*** is our ancestral abundance and modern prosperity, again for the individual and the collective, represented by the imagery of freshwater, the source of our life and wealth. The western cardinal direction aligns with the Hawaiian orientation of facing the west, the ancestral abundance of our past. In honoring the wisdom of those who came before, we envision a future for our *keiki* of concrete wealth (career and professional *ʻauwai* or pathway with sustainable wages) in the context of true cultural wealth (personal fulfillment, pride in identity, work that serves *ʻāina*, and health/education/financial systems that honor our cultures and traditions).
- ***Pilina*** are our mutually sustaining relationships based on our values and principles. The imagery of woven *lau hala* sails signifies the canoe and ocean as our connections to our broader *ʻohana*. The southern cardinal direction honors our original inhabitants of the archipelago arriving from the south, maintaining our connection with tradition and the ancestors.
- ***Ea*** is individual and collective self-determination and agency. The imagery of wind or air represents our *kaiāulu*.

*The word “*ea*” has several meanings. As Hawaiian language and political scholar Leilani Basham argues, each utterance of the word carries all these meanings at once, even when one meaning may be emphasized.* Ea *refers to political independence and is often translated as “sovereignty.” It also carries the meanings “life,” “breath,” and “emergence,” among other things. A shared characteristic in each of these translations is that ea is an active state of being. Life breathing,* ea *cannot be achieved or possessed; it requires constant action day after day, generation after generation*

[48].

The traditional orientation of Hawaiian navigators faced the western horizon, thus the position at east suggests that *ea* is always at our backs, the winds of emerging times, pushing our canoe.

## 5. Conclusions

The Kaiona Framework presented here is a living, evolving effort. Our collaboration within the ʻAha Kū community council will center on how we as a collective can embody and perpetuate the communal values of the framework in our communities: holistic health, connections and relationships, sustainable prosperity, and self-determination.

The Kamaehu o ke Kaiāulu effort continues on, rooted in the collective vision of the ʻAha Kū as represented by the Kaiona Framework. Firstly, we are creating quantitative and qualitative methodologies based on the Kaiona Framework to more accurately assess the well-being of our youth and impact of community efforts. We are working with partners in the healthcare, education, and nonprofit sectors to both develop these approaches and explore how they can augment and even replace current measurement strategies that fail to align with local priorities and values. Secondly, we will use these methodologies to evaluate the impact of Kaiona Programs, efforts already taking place in our community directed at supporting youth. Our work is uplifting these efforts through a formal referral system from our health center that will be assessed in clinical trials. Lastly, we will track the broader reach and impact of the Kaiona Framework as it is shared across our community and beyond. We as a collective have already begun using the framework as a lens to enhance planning and evaluation around our internal operations. At the same time, the framework has become a powerful outward-facing tool to shape the broader narrative surrounding our community.

We acknowledge that this is a living framework and that revisions, additions, and new insights will arise as our work progresses. In this process, we hope to uplift the inherent wealth, gifts, and resilience of our youth while dismantling harmful narratives of poverty, ecological destruction, and social inequities. The idea of innovations that are culturally and communally informed is the only means to transformative and long lasting change for the *ʻāīna*, for the *kaiāulu*, and for the *hōnua* (Earth). We have a very special opportunity to embody this principle and allow Kaiona to guide us to *aloha ʻāina, ʻāīna aloha* at the horizon.

“E lauhoe mai nā waʻa; i ke kā, i ka hoe; i ka hoe, i ke kā; pae aku i ka ʻāina.

*Everybody paddle the canoes together; bail and paddle, paddle and bail, and the shore is reached.*

Pitch in with a will, everybody, and the work is quickly done

([1], #327).”

## Figures and Tables

**Table 1:** Sampling of existing frameworks outside of Hawaiʻi.

Well-Being Framework	Place of Origin	Relevance to Local Context as Identified by Investigators
He Ara Waiora [25] Pacific Wellbeing Strategy [26] The Living Standards Framework [27]	Aotearoa (New Zealand)	Model of culturally grounded perspectives accepted and codified at highest levels of governance
Gross National Happiness Index [28]	Bhutan	Model of widely accepted holistic perspective on well-being with wide evidence base and long-standing relevance
Donut Economics [29]	United Kingdom	Novel approach of lower and upper limits on desired goal domain as model of sustainability and balance
The Flourish Measure [30]	USA	Model of quantifying a broad, holistic approach to well-being

**Table 2:** Sampling of existing frameworks within Hawaiʻi.

Framework	Creators	Advantages Identified by ʻAha Kū Reviewers
Resilience Model of Adult Native Hawaiian Health	Antonio et al. [31]	- Data-driven focus of this model - A scientific way to articulate and quantify resilience - Replicable for different contexts (i.e., can measure things other than resilience)
Ahupuaʻa Framework	Daniels et al. [32]	- Ties into cultural aspects - Visual layout is beautiful
Nā Hopena Aʻo or HĀ	Hawaiʻi Department of Education [33]	- How it focuses on you, the person, in order to contribute to the community - The *ʻŌlelo* (utilization of Hawaiian language) - Concept centered around Hawaiian values, multidisciplinary - *Inoa* (name) concept - Incorporation of *ʻŌlelo noʻeau* (traditional Hawaiian proverbs)
Nā Pou Kihi	Kaholokula; Native Hawaiian Health Task Force, Force et al. [34,35]	- The clarity and specificity of the domains of Native Hawaiian health
The Pua Model: A Native Hawaiian Perspective on Well-Being	Kamehameha Schools [36]	- Focus on holistic domains - Inclusion of economics and material well-being
Pilinahā: An Indigenous Framework for Health	Kōkua Kalihi Valley Comprehensive Family Services, Odom et al. [37]	- Emphasis on connections; health has a direct link to feeling connected - Place-based model, clear understanding and respect for place - Visually interesting shape - Simplicity of this model, open for interpretation - Centering on Indigenous health, not necessarily just Native Hawaiians
Kūkulu Kumuhana	Lili’uokalani Trust [38]	- Clear, visually attractive, simple graphics - Key components of Hawaiian concepts - Six dimensions are balanced, one does not supersede the other - Framework is simple
An Ecological Model of Native Hawaiian Well-being	McGregor et al. [5]	- Good flow on well-being - Encompasses all aspects (individual, ʻohana, community, etc.) - Model set foundation for future models

**Table 3:** Guiding principles for the development of a place-based, Waiʻanae-specific framework.

Driving Principles
The framework calls people home, back to themselves, back to their identity—that is the essence of this work. The point of this study is to dismantle mis-narratives, harmful narratives. It is measurable. The framework catalyzes those outcomes. The essence remains constant, the form is in flux. The pursuit of values is what to measure, and outcomes will show themselves. Evaluation and methods should all be rooted in observation. The storytelling *is* measurement.
**Elements**
Foundation in *moʻolelo* (traditional stories) with which our community identifies Framework of practice as valuable as a framework of outcomes Consideration of outcomes that matter now (e.g., conflict resolution) Grounding in principles adopted by ʻAha Kū Kamaehu Importance of economics
**Desired Features**
Cultural relevance: Tie to *moʻolelo*; not limited to one culture; inclusion/prioritizing *ʻāina* (natural environment) Simplicity: Relatability to community; clear graphics, readily understandable, and also open to individual interpretation Quantifiable Practicality Adaptability (not prescriptive) Place-based Community input incorporated Connections: health, place, beyond individual level
**Features to Avoid**
Top-down, prescriptive approach Overly academic, out-of-touch with community Unapproachable, overly complicated graphics Something that would be temporary or limited in its use

## Data Availability

The datasets presented in this article are not readily available because of the sensitive cultural and personal nature of qualitative interviews, focus groups, and meetings. Requests to access the datasets should be directed to the corresponding author.

## Abbreviations

The following abbreviations are used in this manuscript:

NIH	USA National Institutes of Health
WCCHC	Waianae Coast Comprehensive Health Center
CBO	Community-Based Organization
